# *Bacillus amyloliquefaciens*- modified clay for controlling harmful algal blooms in eutrophic aquaculture ponds

**DOI:** 10.3389/fmicb.2025.1550905

**Published:** 2025-03-06

**Authors:** Zaixing Wu, Zhiming Yu, Xiuxian Song, Kaiqin Jiang, Xihua Cao, Yongquan Yuan

**Affiliations:** ^1^CAS Key Laboratory of Marine Ecology and Environmental Sciences, Institute of Oceanology, Chinese Academy of Sciences, Qingdao, China; ^2^Ocean Decade International Cooperation Center (ODCC), Qingdao, China; ^3^Laboratory for Marine Ecology and Environmental Science, Qingdao Marine Science and Technology Center, Qingdao, China; ^4^Center for Ocean Mega-Science, Chinese Academy of Sciences, Qingdao, China; ^5^College of Marine Sciences, University of Chinese Academy of Sciences, Beijing, China

**Keywords:** harmful algae blooms (HABs), microbial modified clay, *Bacillus amyloliquefaciens*, healthy aquaculture ecosystem, HABs control, *Heterosigma akashiwo*

## Abstract

**Introduction:**

*Bacillus amyloliquefaciens* is a commonly used probiotic microecological regulator in aquaculture water with the ability to inhibit harmful algae blooms (HABs) and improve the health status of aquaculture ecosystem. Modified clay has been widely applied in the field for eliminating HABs.

**Methods:**

In this study, clay particles are used as carriers and to provide a microenvironment for microbial fermentation, yielding a microbial modified clay product with high efficiency for eliminating typical HAB organisms. Methods are developed for the fermentation of microorganisms and clay particles and also for subsequent preparation of a dry powder, which stably produce microbial modified clay in dry powder form for HAB elimination.

**Results:**

The results showed that the obtained microbial modified clay has a stable bacterial content of more than 10^9^ cfu/mL, and the removal rate of the HAB organism *Heterosigma akashiwo* equivalent to that obtained with other HAB removal materials at the same dosage, indicating good potential for HAB removal. The removal rate of *Heterosigma akashiwo* at a dosage of 0.1 g/L microbial modified clay exceeded 90%.

**Discussion:**

By combining two natural and commonly used material, both the function of emergency flocculation disposal (when HAB outbreaks) and long-term ecological regulation (to maintain a healthier phytoplankton community structure through specific algicidal effect) were achieved, resulting in a 1 + 1 > 2 effect when applied in the eutrophic aquaculture environment. Also, this exploratory study with the unique combination of microbial fermentation and clay modification preliminarily provides an important scientific basis for promoting the large-scale application of microbial modified clay in the field of HAB control, especially in the eutrophic aquaculture ecosystems. Also, extensive ecological assessment before field application is still required, such like the scientific support of environmental criteria, the safety to other aquaculture organisms and the ecological effect of the whole aquaculture ecosystem.

## 1 Introduction

Bacteria and phytoplankton (e.g., single-cell microalgae) in terrestrial freshwater and marine ecosystems exhibit a complex and dynamic relationship (Coyne et al., 2022). The “bacteria–algae” relationship involves a series of complex physical, biological and chemical processes (Zhou et al., 2014). Through these processes, bacteria and phytoplankton jointly influence and promote biogeochemical cycling in terrestrial freshwater and marine ecosystems, maintaining ecological balance in the aquatic environment (Jung et al., 2010; Rediat et al., 2024). Harmful algal blooms (HABs), also known as red tide in coastal waters, occur frequently worldwide (Yu and Chen, 2019; Anderson et al., 2022). As typical marine disasters in eutrophic environment, HABs cause considerable economic losses and harm inshore/offshore aquaculture ecosystems and human health (Chen, 2018; Neves et al., 2021).

Bacteria that induce algal lysis in HAB organisms, called algicidal bacteria, have been recognized and isolated during HAB events. These bacteria include the Cytophaga/Flavobacterium/Bacteroidetes (CFB) group and the γ-proteobacteria group, e.g., the genera *Alteromonas, Bacillus, Cellulophaga, Cytophaga, Flavobacterium, Micrococcus, Planomicrobium, Pseudoalteromonas, Pseudomonas, Saprospira, Vibrio, and Zobellia* (Demuez et al., 2015). The mechanisms of algicidal effects are complex and diverse and generally involve two processes: direct contact of bacteria with HAB cells and indirect excretion of active algicidal compounds that cause algal lysis (Mayali and Azam, 2004; Jesús et al., 2024). The latter indirect process is the main mode of action for most algicidal bacteria (Huang et al., 2019); moreover, the structural characteristics and functional mechanisms of confirmed algicidal compounds are highly diverse (Meyer et al., 2017).

As a typical biological prevention and control method, algicidal bacteria have become a widely explored topic in the field of HAB mitigation and control in terrestrial and coastal waters. Scientists have conducted effective research on the screening of algicidal bacteria; the interactions between algae and bacteria; the effects of algicidal bacteria on the cell structure, physiological and biochemical stress of algae; and the functional gene expression changes in algae cells under controlled conditions (Zheng et al., 2011; Li et al., 2016; Zhou et al., 2016; Zheng et al., 2018). However, current research on the biological control of HABs using algicidal bacteria is mostly in the experimental stages in the laboratory (Pal et al., 2020). Only a few studies have been conducted in open water, such as an assessment of the algicidal effect of *Pseudomonas fluorescens* in a field study in river enclosures (Noh et al., 2017). Many biotic and abiotic factors hinder the large-scale application of algicidal bacteria as a biological prevention and control method in the field. For example, although some bacteria have exhibited strong algicidal effects on HAB organisms in laboratory culture experiments, the effects in uncontrolled field environments are often unstable (Jung et al., 2010; Kang et al., 2011). In field applications, algicidal bacteria often fail to reach the minimum concentration threshold for algae lysis, or high concentrations of algicidal compounds can be diluted immediately by external water bodies, resulting in significantly weakened algicidal effects in HAB control (Jeong and Son, 2021). The biosafety of algicidal bacteria has also received widespread attention; for example, *Vibrio brasiliensis*, which has good algicidal effects on HAB organisms (Ouyang et al., 2021), also exhibits pathogenicity to cultured biological shrimp (Li et al., 2021). The above reasons collectively explain why algicidal bacteria have not been widely used in the field as a biological control method for HABs.

Among the current HAB mitigation and control methods in the field, modified clay flocculation is characterized by environmental friendliness, a high removal efficiency, and a quick elimination effect. This method has been applied at a large scale in the field to control HABs (Yu et al., 2017). In this approach, the flocculation between natural clay and HAB organisms is enhanced through modification of the clay surface, thereby significantly reducing the amount of clay used and significantly improving the efficiency of HAB flocculation and control. The current mature modification methods include inorganic modification, organic modification, and inorganic–organic composite modification (Cao et al., 2006; Zhang et al., 2013; Liu Y. et al., 2016). Although most commonly used in the fields, the setbacks of inorganic and organic modified clays include that they are merely HABs emergency disposal material when HAB outbreaks and they lack the functional attributes of long-term regulations of water quality or microalgae community, which is severely needed by the aquaculture ecosystems.

Several difficulties in translating algicidal bacteria application from the laboratory to the field still need to be addressed; as such, microbial modified clay might represent a promising, green and safe HAB control technology. The rationale for developing Microbial modified clay is simply because it combines clay, which is already widely used in the treatment of HABs, with commonly used functional microorganisms. The combination will result in the function of both emergency flocculation disposal and long-term ecological regulation when applied in the eutrophic aquaculture environment. A few studies have combined clay particles and widely used probiotics in marine aquaculture environments. Liu et al. (2021) further improved the removal efficiency of HAB organisms by combining effective microorganism (EM) bacteria with clays. Zhong et al. (2022) conducted an experiment exploring a biologically modified clay prepared by combining *Bacillus amyloliquefaciens* and kaolin clay in laboratory, and the results further enriched the variety of potential biologically modified clay materials suitable for controlling HABs.

On the basis of previous laboratory research on the removal of HAB organisms by microbial modified clay, this study preliminarily explored the large-scale fermentation conditions and parameters for the preparation of microbial modified clay, as well as the production steps from the fermentation broth phase to the microbial modified clay powder phase for field application. The removal efficiency of typical HAB species by the obtained microbial modified clay was also evaluated through HAB removal experiments. We hope that this study will provide an important scientific reference for promoting trial applications or large-scale field applications of microbial modified clay for HAB control after further ecological assessment in the future.

## 2 Materials and methods

### 2.1 Experimental materials

The microorganism used to prepare the microbial modified clay powder in this study was *Bacillus amyloliquefaciens* (bacterial strain MH210892.1), which was purchased from Qingdao Shangde Biotechnology Co., Ltd. The strain was first revived and cultured in Luria-Bertani (hereafter LB) liquid medium for 24 h, after which it was separated and purified by agar plating to obtain purified *Bacillus amyloliquefaciens*.

The clay material used to prepare the microbial modified clay was kaolin clay from BINTANG PUSPITA BUMIDWIPA Co., Ltd., Indonesia. The clays are tiny powder with an average particle size of 6.8 μm and a surface potential of −8.18 ± 0.51 mV. The main contents of the clay include SiO_2_ (about 45 %), Al_2_O_3_ (about 31%), K_2_O (about 1.8%). Fe_2_O_3_ (about 0.8%), CaO (about 0.1%), MgO (about 0.2%) and is a green, natural, environmental friendly material.

As a typical HAB organism commonly observed in the coastal waters of China and around the world, the HAB species *Heterosigma akashiwo* was often observed as the dominate HAB species in the aquaculture ponds in China coastal areas. When HABs of *Heterosigma akashiwo* outbreaks, the water of the ponds exhibited some color of “soy sauce” and caused great economic losses to the aquaculture industry. So, in this study, *Heterosigma akashiwo* was used in the removal experiment and was obtained from the HAB species bank of the Key Laboratory of Marine Ecology and Environmental Sciences, Chinese Academy of Sciences. Compared with other species such as P*rorocentrum donghaiense and Karenia mikimotoi*, this HAB species is not easily eliminated by traditional inorganic modified clays. The algae were cultured in L1 medium at 20 ± 1°C, a light intensity of 72 μmol photons/(m^2^⋅s) and a light to dark ratio of 12:12 h. In the initial stage of cultivation, the solution was inoculated with algae in the exponential growth phase and regularly shaken to promote algal growth. After the algae reached the plateau stage and exceeded the commonly believed HAB outbreak density of 1.0 × 10^5^ cells/mL or above, the solution was used in experiments on the removal efficiency of HAB organisms by microbial modified clay.

### 2.2 Fermentation process and production of microbial modified clay

#### 2.2.1 Cultivation of fermentation starter culture

In a sterile environment, an inoculation ring was used to transfer a single purified *Bacillus amyloliquefaciens* colony into a Kjeldahl flask containing a solid LB culture medium slant, and the flask was immediately sealed with a cotton stopper wrapped around the bottle mouth. After 48 h of incubation in a 30°C constant-temperature incubator, bright white and clear colonies were spread on the slant of the solid culture medium inside the Kjeldahl flask, and these colonies were used as the liquid fermentation starter culture for inoculation in a 50 L fermentation tank. The starter culture was resuspended in sterilized physiological saline and then inoculated into a fermentation tank under sterilized conditions for 24 h of fermentation.

#### 2.2.2 Preparation of fermentation medium

Approximately 32 L of water was added to the 50 L tank, and after air sterilization, an additional 3 L of condensed water was generated to obtain a total 35 L of broth. Unlike previous microbial fermentation procedures that used only specific culture media in the tank, this study selected both culture medium and kaolin clay as composite fermentation materials in the 50 L fermentation tank. The stirrer was started to evenly mix the culture media mixture, after which the pH of the solution was adjusted to 7.0 ± 0.2 with sodium hydroxide.

As a preliminary study, we choose the most commonly used LB culture medium for microorganisms as the fermentation medium. Furthermore, our previous laboratory-scale shake flask fermentation process also revealed that the resulting fermentation broth of *Bacillus amyloliquefaciens* by LB culture medium had a good removal effect on HAB organisms (Zhong et al., 2022). The final concentration of each substance in the fermentation tank was 10 g/L peptone, 5 g/L yeast powder, 5 g/L sodium chloride, and 25 g/L kaolin clay.

#### 2.2.3 Optimal fermentation and spray drying parameters

After the fermentation materials were added to the fermentation tank, the pH was adjusted to 7.0, and the mixture was sterilized at a high temperature of 121°C for 30 min. After sterilization and when the temperature of the culture medium decreased to 30°C, *Bacillus amyloliquefaciens* starter materials were scraped from the Kjeldahl flask, resuspended in physiological saline, and transferred to the 50 L fermentation tank for fermentation and cultivation. The cultivation conditions included a temperature of 30°C, stirring speed of 300 rpm, sterilized air ventilation rate of 1:1, tank pressure of 0.05 MPa, and dissolved oxygen concentration higher than 20%. If the dissolved oxygen concentration was insufficient, the above parameters could be adjusted to 650 rpm, 0.08 MPa of pressure, and the 1:1.5 of ventilation rate. Fermentation was stopped after 24 h.

A centrifugal spray drying tower was used to dry samples of fermentation liquid collected at four different fermentation times (6, 10, 18, and 24 h) to obtain microbial modified clay in dry powder form. The spray drying operating parameters were as follows: inlet air temperature of 175°C; atomizer frequency of 40 Hz; and air outlet temperature of 78–80°C. The microbial modified clay powder obtained through this process had a low water content and could be stored for a long time without caking. To compare the differences between this spray drying method and other drying methods, liquid nitrogen freeze-drying was also performed for comparison. In this method, 50 mL of microbial modified clay suspension was centrifuged at 8819 × *g* for 10 min, and the precipitate was frozen in liquid nitrogen for 5–15 min; then, the powder was frozen and dried under vacuum at a low temperature (approximately −50°C) for 24 h, ground with a mortar, passed through a 200 mesh sieve, dried and stored for use.

### 2.3 HAB organism removal experiment

The removal effect of the microbial modified clay on a typical HAB alga was tested by mixing a certain amount of microbial modified clay powder with deionized water to create a modified clay stock suspension, which was then sprayed and added onto algal solution surface containing the red tide alga *Heterosigma akashiwo*. After treatment for a certain period, samples were collected from the upper layer of the algal solution to measure the changes in algal cell density before and after the addition of the microbial modified clay. Specifically, *Heterosigma akashiwo* was cultured (approximately 10^5^ cells/mL) until the middle and late stages of exponential growth, after which the solution was added to 50 mL colorimetric tubes. The above algal solution samples were divided into experimental and control groups, with three parallel samples in each group. After a certain amount of microbial modified clay was adequately mixed with deionized water, it was sprayed and added onto the algae solutions in the experimental groups and shaken thoroughly. The concentration of microbial modified clay added to the algal solution was the same as that use for several other red tide elimination materials tested in this study, including natural clay, modified clay type I and type II (MCI and MCII, these two are previously produced inorganic Aluminum based modified clays by our lab), directly purchased commercial bacteria (this is a production by Qingdao Shangde Biotechnology Co., Ltd., and it only include one bacterial taxon of *Bacillus amyloliquefaciens*), and a simple mixture of commercial bacteria and natural clay (ratio of 1:1); all materials were added at a concentration of 0.2 g/L. The algal solutions in the control group were not subjected to any treatment. In addition, the removal rate of *Heterosigma akashiwo* in the dosage-removal effect experiment was also investigated at different concentrations of microbial modified clay (five concentrations: 0.05, 0.1, 0.2, 0.3, and 0.5 g/L). Samples were taken at 3 and 24 h to measure the chlorophyll *in vivo* fluorescence of residual algal cells in the supernatant after suppression and sedimentation of elimination materials in the colorimetric tubes of the experimental and control groups, respectively. The algal removal efficiency of the different materials is expressed by the removal rate, which is calculated as follows (Formula 1): Algicidal rate (%) = (1–experimental algal density/control algal density) × 100%

### 2.4 Mechanism of algae removal by bacteria

To test the algal removal mechanism of the microbial modified clay, three different algal removal experiments were carried out: (1). Deionized water was used to prepare a stock solution of the microbial modified clay powder, and the stock solution was directly added to *Heterosigma akashiwo* immediately; (2). The above stock solution was centrifuged (3,824 × *g*, 10 min) to obtain the precipitate and supernatant, the precipitate was washed three times with deionized water, and the precipitate was finally resuspended with an equal volume of deionized water to obtain a bacteria and clay resuspension; (3). The supernatant was filtered with a 0.22 μm filter membrane three times to obtain sterile filtrate. One milliliter and 2.5 mL of each of the above three elimination materials were added to 50 mL of *Heterosigma akashiwo* to conduct the algae removal experiments described above. The final addition concentrations were 0.2 and 0.5 g/L. The samples were mixed well, and after the 24 h experimental period, the chlorophyll *in vivo* fluorescence was measured to calculate the removal rate via formula (1).

### 2.5 Measurement methods for other parameters

To assess other relevant parameters in the fermentation and cultivation process, the live bacterial biomass in the composite microbial clay fermentation broth was monitored, and growth curves were calculated. After the start of cultivation, 50 mL samples were taken every 2 h and immediately microscopically examined and counted to determine the bacterial growth, death, spore formation, and cell count and the growth curve. The viable bacterial content of the microbial modified clay was also determined. In addition to conducting sampling every 2 h to monitor growth, we removed fermentation broth samples at four times (6, 12, 18, and 24 h) to obtain microbial modified clay powder; additionally, the number of bacterial cells was counted in the microbial modified clay powder samples obtained after centrifugation spray drying of fermentation broth samples collected at 6, 12, 18, and 24 h.

In the above experiments, counting was performed as follows: for the fermentation broth, the broth was extracted, and the number of bacteria (cfu/mL) was estimated. The solution was diluted 10^–7^ times with sterilized physiological saline. A total of 0.1 or 0.05 mL of the 10^–6^ or 10^–7^ dilution was streaked onto an LB solid plate. The plate was placed in a 30°C constant-temperature incubator for 24 h until a clear single plaque grew, and the number of colonies was calculated (for quality control purposes, the number ranged from 30 to 300). For the dry powder, a certain amount was weighed, mixed with glass beads, sterilized with physiological saline, shaken and activated for 20 min. The same steps were then used to determine the total bacterial count of the dry powder (cfu/g).

## 3 Results

### 3.1 Impact of fermentation time on the biomass of bacteria in microbial modified clay

#### 3.1.1 Growth curve of bacteria in the fermentation broth

**FIGURE 1:**
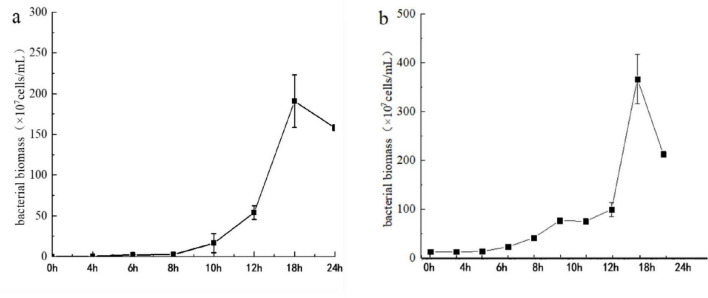
Growth curves of bacteria in the microbial clay fermentation broth during two fermentation processes conducted in different times. Left line in panel **(a)** represent growth curve cultivated on 24^th^ Apr, 2022 and right line in panel **(b)** represent growth curve cultivated on 8^th^ Jul, 2022.

Monitoring of the live bacterial biomass in the microbial clay fermentation broth over 24 h showed (Figure 1) that the initial inoculation density of bacteria in the entire fermentation system was approximately 10^6^ cfu/mL. As the fermentation time increased, the bacterial density in the fermentation broth gradually increased, reaching 10^9^ cfu/mL after 12 h. The density remained above 10^9^ cfu/mL until the end of fermentation. As the fermentation time increased, with the consumption of culture medium as well as other factors, the bacterial content of the fermentation broth in the liquid LB and clay formula slightly decreased after reaching its peak at approximately 18 h but remained above 10^9^ cfu/mL.

#### 3.1.2 Changes in bacterial biomass in the microbial modified clay at different fermentation times

The bacterial biomass (in cfu/g) of microbial modified clay powder obtained using spray drying was monitored four different times during fermentation (6, 12, 18, and 24 h). The results indicate that the duration of fermentation is positively related to the bacterial biomass of the final microbial modified clay powder (Table 1). As the fermentation time increased, the number of live bacteria (including spores) in the final microbial modified clay powder gradually increased. After 24 h of fermentation, the microbial biomass of the dry powder obtained by spray drying reached 4.13 × 10^9^ cfu/g. To better compare the difference between the number of live bacteria in the fermentation broth (including spores, cfu/mL) at different time points and the number of live bacteria (including spores, cfu/g) in the spray-dried powder at that same time point, the bacterial biomass of the spray-dried powder was converted from cfu/mL to cfu/g to obtain the theoretical value; this value corresponds to the ideal case with no loss of live bacteria during the high-temperature centrifugal spray-drying process. This calculation is based on the actual 1.5 kg dry powder weight obtained from 35 L of fermentation broth in each tank in the spray drying process. Finally, the theoretical conversion ratios (the ratio of bacterial biomass in the actual spray-dried powder to the theoretical bacterial biomass directly calculated from the fermentation broth) corresponding to the four different time points were obtained (Table 1). The results showed that although the number of live bacteria in the fermentation broth decreased at 24 h, the dry powder conversion ratio was the highest after spray drying. However, although the number of live bacteria in the fermentation broth reached its peak in the early stage at hour 18, the final dry powder conversion ratio at this time was very low.

**TABLE 1:** Number and ratio of bacteria in Luria-Bertani (LB) culture medium (fermentation broth) and spray-dried powder at different fermentation times.

FT	NOB1	NOB2	TCR1	TCR2
6 h	4.2 × 10^6^ cfu/g (σ: 3.7 × 10^5^)	1.8 × 10^7^ cfu/mL (σ: 2.1 × 10^6^)	4.2 × 10^8^ cfu/g	1% (low total amount)
10 h	6.4 × 10^6^ cfu/g (σ: 1.2 × 10^6^)	3.8 × 10^8^ cfu/mL (σ: 3.2 × 10^7^)	8.7 × 10^9^ cfu/g	0.07%
18 h	1.2 × 10^7^ cfu/g (σ: 9.2 × 10^5^)	3.9 × 10^9^ cfu/mL (σ: 7.0 × 10^8^)	9.1 × 10^10^ cfu/g	0.01%
24 h	4.1 × 10^9^ cfu/g (σ: 9.5 × 10^8^)	1.2 × 10^9^ cfu/mL (σ: 9.0 × 10^8^)	2.8 × 10^10^ cfu/g	14.6%

FT, fermentation time; NOB1, number of bacteria (including spores, cfu/g) in the spray-dried powder; NOB 2, number of bacteria (including spores, cfu/mL) in the fermentation broth; TCR1, theoretically calculated bacterial biomass in ideal dry powder from fermentation broth without loss; TCR2, theoretical conversion ratio: bacterial biomass in the spray-dried powder/converted theoretical bacterial biomass calculated from the fermentation broth.

### 3.2 Removal effect of microbial modified clay on typical HAB organisms

#### 3.2.1 Effect of microbial modified clay prepared at different fermentation times on *Heterosigma akashiwo* removal

**FIGURE 2:**
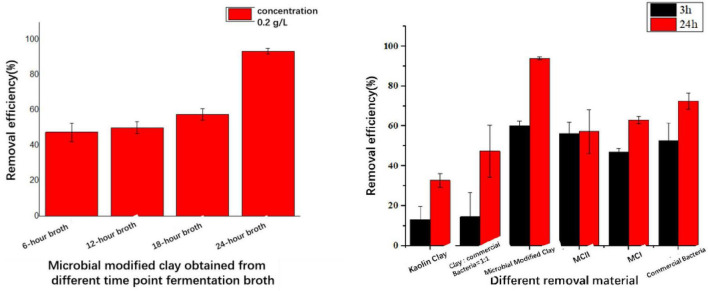
**(left)** Comparison of the removal effects of microbial modified clay in dry powder form at different fermentation times (6/10/18/24 h) on the red tide organism *Heterosigma akashiwo*. **(right)** Comparison of removal efficiency between microbial modified clay (24 h fermentation) and other types of modified clay. Inorganic modified clays (MCI and MCII) are two previously produced inorganic Aluminum based modified clays by our lab.

Fermentation broth samples collected at 6, 10, 18 and 24 h were dried with a centrifugal spray drying tower to obtain microbial modified clay in dry powder form. Then, the removal effect of the resulting powders was tested on algal solution containing the HAB organism *Heterosigma akashiwo* at a dosage of 0.2 g/L microbial modified clay and an algal density of 10^6^–10^7^ cells/L (as shown in Figure 2 left). Additionally, to further evaluate the removal effect of the microbial modified clay material, the algal removal rate of the final dry powder (at 24 h fermentation time) was compared with those of the other HAB removal materials (Figure 2 right and Table 2). The calculated removal rates [see Formula (1)] over a 1 day removal cycle (24 h) revealed that the longer the fermentation time of the fermentation broth was, the greater the removal effect of the microbial modified clay on *Heterosigma akashiwo*. The trend in the removal effect is consistent with the variations in the bacterial biomass of the microbial modified clay samples obtained at different fermentation time points. The microbial modified clay products obtained from fermentation times of 6 and 10 h had relatively weak removal effects on the red tide algae. After 18 h of incubation, as the bacterial biomass increased with fermentation, the bacterial biomass of the dry powder reached 1.2 × 10^7^ cfu/g, which was already an order of magnitude higher than those at 6 and 10 h of incubation. After 24 h of treatment, the algal removal effect of the microbial modified clay exceeded the removal rate of the inorganic modified clay (MCI) at the same dosage. The microbial modified clay obtained after 24 h of fermentation/incubation, when added at a concentration of 0.2 g/L, had a *Heterosigma akashiwo* removal rate of over 90% after 24 h of treatment, which is a relatively high removal rate of HAB organisms.

**TABLE 2:** Comparison of removal efficiency between microbial modified clay and other types of modified clay.

Types of HAB eliminator	Average RE (3 h)	Average RE (24 h)
Kaolin clay	12%	33%
Clay: commercial bacteria	13%	46%
Microbial modified clay	59%	93%
MCI	49%	60%
MCII	56%	57%
Commercial bacteria	51%	71%

RE means removal efficiency.

Compared with the effects of the other red tide removal materials added at the same total concentration (0.2 g/L), the effect of the inorganic modified clay was faster (removal reached approximately 50% after 3 h of treatment), indicating the speed advantage of traditional inorganic modified clay. However, the increase in removal rate in the later stage (from 3 to 24 h of treatment) was less than that of the microbial modified clay. In addition, the *Heterosigma akashiwo* removal rate of the microbial modified clay was significantly higher than that of commercial *Bacillus amyloliquefaciens* (produced via fermentation without clay particles, with a very high bacterial content of 100 billion per gram) and was also higher than that of natural kaolin clay, the two tested types of traditional inorganic modified clays (MCI, MCII), and the simple mixture of clay and commercial *Bacillus amyloliquefaciens* (mass ratio of 1:1). The microbial modified clay material produced via the above fermentation process had the same composition as the simple mixture of clay and commercial *Bacillus amyloliquefaciens*. However, the produced microbial modified clay had a significantly higher removal rate than the simple mixture of the two materials, even though the latter had a higher biomass.

#### 3.2.2 Removal effect of microbial modified clay powder prepared via different drying methods on the HAB organism *Heterosigma akashiwo*

**FIGURE 3:**
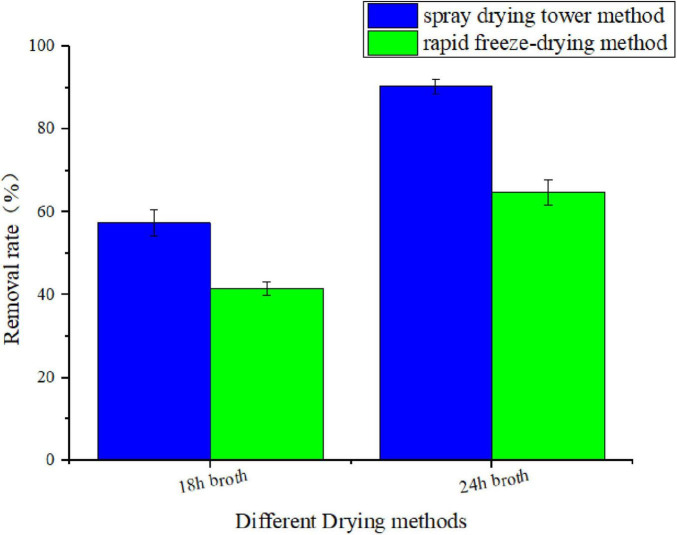
Removal rate of the red tide organism *Heterosigma akashiwo* by microbial modified clay prepared at 18 and 24 h of fermentation via rapid freeze-drying and tower spray drying (removal rate of red tide algae 24 h after the addition of microbial modified clay).

To explore the optimal preparation parameters for the microbial modified clay, a laboratory-scale low-temperature freeze–drying method with liquid nitrogen was tested in addition to the high-temperature spray–drying tower method used in this process to compare the removal effects of the two dry powders on the HAB organism *Heterosigma akashiwo* (as shown in Figure 3). The results showed that the removal efficiency of the microbial modified clay powder obtained by high-temperature spray drying was approximately 30%–40% higher than that of the modified clay obtained by laboratory low-temperature rapid freezing with liquid nitrogen when either the microbial modified clay powder obtained at 18 or 24 h of fermentation was used.

#### 3.2.3 Dosage-removal effect of microbial modified clay on *Heterosigma akashiwo*

**FIGURE 4:**
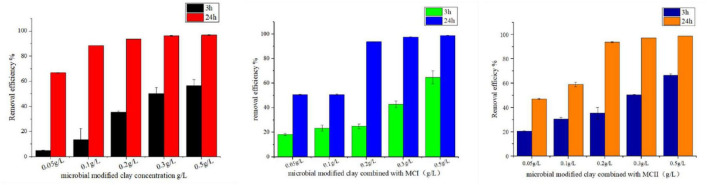
Removal effect of microbial modified clay products on red tide algae at different concentrations [**(left):** microbial modified clay; **(center):** microbial modified clay combined with inorganic modified clay (MCI); **(right):** microbial modified clay combined with inorganic modified clay (MCII)].

The effect of different final concentrations/doses of microbial modified clay on the removal rate of the red tide organism *Heterosigma akashiwo* was investigated (Figure 4 left). The results showed that after 3 h of treatment, the removal rate of the microbial modified clay was directly proportional to the clay concentration. After 24 h, with the exception of the addition concentration of 0.05 g/L, the removal rate of the other addition concentrations (0.1–0.5 g/L) exceeded 90%, which was better than that of inorganic modified clay at the same dosage. By combining the microbial modified clay with inorganic modified clay, the amount of the former material required for HAB removal was further reduced (Figures 4 centre, right). With a concentration of 0.1 g/L microbial modified clay and a total addition amount of 0.2 g/L, the removal of red tide algae exceeded 90% (the efficiency of HAB removal was determined by concentration of the bacteria and the biomass of the HAB cells, see more in the discussion part). Moreover, in the combined approach, the dosage of the traditional high-efficiency inorganic modified clay was also reduced by 50% (from 0.2 to 0.1 g/L MCI and MCII), while its rapid elimination effect may be fully realized, achieving a synergistic advantage in algae removal.

#### 3.2.4 Possible functional components of algae removal by bacteria

**FIGURE 5:**
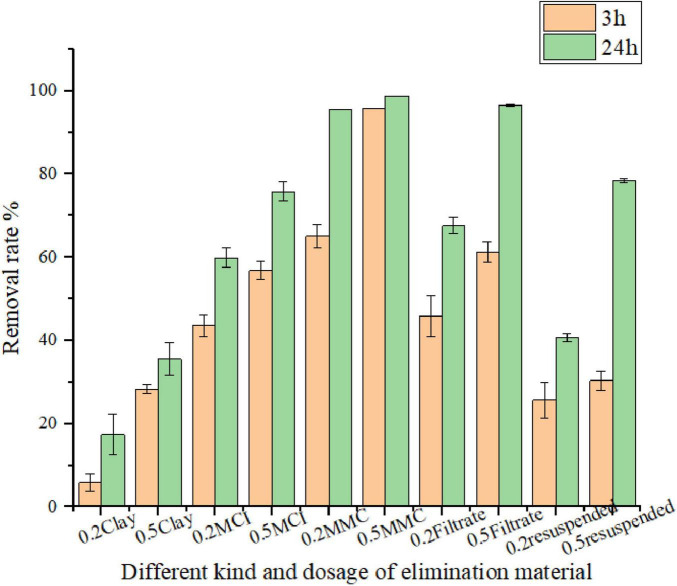
Removal efficiency of different components of *Bacillus amyloliquefaciens* fermentation broth on *Heterosigma akashiwo*. MMC refers to the microbial modified clay.

Bacterial modified clay powder contains various components, such as bacterial cells, clay particles, culture media, and bacterial secretions known as algicidal substances or algicides. To identify the main algae removal components in the bacterial solution, we also investigated the removal effects of various components, such as the microbial modified clay, bacteria and clay resuspension, and filtrate, on red tide organisms. The results after 3 and 24 h of reaction between each component and the red tide algae solution are shown in Figure 5. The results demonstrated that the filtrate (algicidal substances) had a greater effect than other bacterial cell components. After 24 h, the removal rates of *Heterosigma akashiwo* by the microbial modified clay stock solution were 95% and 98% in the 0.2 and 0.5 g/L groups, respectively, whereas the removal rates of the filtrate reached 67% and 96%, respectively. The removal effect of the bacteria and clay resuspension on algae cells was relatively weak, with removal rates of 40% and 76%, respectively. At 3 h, the removal rate was much lower, with values of approximately 20% and 30% in the 0.2 and 0.5 g/L groups, respectively, which were almost the same as those of the natural clay group. Only after 24 h did the removal rate of the 0.5 g/L group of the suspended clay and bacteria reach a relatively high level, approximately 76%, but the value was still lower than those of the microbial modified clay and the filtrate groups.

## 4 Discussion

### 4.1 Fermentation process and optimal parameters

Although the number of *Bacillus amyloliquefaciens* slightly decreased after 18 h of fermentation, it remained above 1 × 10^9^ cfu/mL, and the spore formation rate increased at 24 h (Table 3). The final live bacterial biomass of the spray-dried powder was also higher at the end of fermentation. At 6 and 10 h, the spore formation rate was extremely low, therefore, the high temperature during the spray drying process led to the death of most microorganisms, resulting in low yields and theoretical conversion ratios, as shown in Table 1. The conversion ratio of dry powder at 18 h after spray drying was still low, probably due to the extremely lower spore formation rate. The spore formation rate and maturity in the 24 h fermentation broth increased, resulting in a significant increase in the yield conversion ratio of dry powder after high-temperature spray drying. Unlike some other fermentation process that reached a spore formation rate of above 90% at 24 h, this study reached approximately 20%–30% spore formation rate, which means more vegetative cells in the broth and might had a higher metabolic activity.

**TABLE 3:** Observations of spore formation during fermentation.

Fermentation time	6 h	10 h	18 h	24 h
Observations of bacterial cells and spores	2–3 bacterial cells in the field of view	Dozens of bacterial cells in the field of view, no spores found	Multiple bacterial cells, occasionally few spores in the field of vision	Approximately 20%–30% spore formation rate and approximately 10% maturity rate

The selection of parameters for fermentation depends on the purpose of the process, that is, the desired biological characteristics of the obtained powder product. The bacterial biomass of our fermentation broth was close to the live bacterial biomass of *Bacillus amyloliquefaciens* in other fermentation processes reported in the literature; however, the spore formation rate was much lower than those reported in the relevant literature (such as a 24 h live bacterial content of 3.56 × 10^9^ cfu/mL and a spore formation rate of 85%, (Xie et al., 2013). In our fermentation process, the system may need more than 24 h to achieve a higher spore formation rate, similar to some studies that also used LB culture medium and reported that more than 72 h was required to achieve a greater than 70% spore formation rate (Wei, 2019). Notably, some fermentation processes are more conducive to obtaining more *Bacillus amyloliquefaciens* cells (especially spores), thereby yielding products with a higher spore content (usually with a spore formation rate greater than 90% and maturity rate greater than 90%); therefore, fermentation medium formulas with a higher live bacterial biomass or higher spore count are desirable. For example, the number of live bacteria and spores in fermented products used in the aquaculture, animal protection, and fertilizer industries is generally in the hundreds of billions (Liu C. et al., 2016), which is much greater than the number of live bacteria in the *Bacillus amyloliquefaciens* fermentation broth in our study.

In contrast, once a large number of spores are formed in the fermentation broth, the bacterial cells enter a dormant state, which reduces their own metabolism and the production of active substances. This may be the reason why a lower removal rate was observed for the commercial bacterial powder than the dry powder obtained by fermentation in our study, although the commercial bacterial biomass was much greater. In our study, the bacterial biomasses of both the fermentation broth at 24 h and the final microbial modified clay powder product were relatively high ( 10^9^ cfu/mL and 4.13 × 10^9^ cfu/g, respectively). In addition, during the entire 24 h fermentation, the spore formation rate did not reach levels as high as those of other processes; this lower rate might be advantageous for algicidal activity since the vegetative cells in the broth had a higher metabolic activity than spore-formation cells. So, the lower spore formation in our study might have promoted the production and secretion of algicidal active substances by live bacteria during fermentation to some extent, thereby yielding a product with both a high biomass of bacteria and a high content of algicidal active substances. In most industrial *Bacillus amyloliquefaciens* fermentation processes, the fermentation duration is controlled at approximately 24 h, which is sufficient to achieve a stable growth curve (Liang et al., 2018). This 24 h period is effective and exhibits good cost performance in terms of live bacterial biomass yield and economic considerations. Additionally, the comparative analysis showed that the microbial modified clay obtained at 24 h of fermentation had the best removal effect; moreover, the microbial modified clay after fermentation had a higher removal rate for the typical HAB organism *Heterosigma akashiwo* than did bacteria alone, as also observed by Zhong et al. (2022), further confirming the suitability of this fermentation process.

### 4.2 Drying process of microbial modified clay derived from fermentation broth

Although the bacterial biomass in the fermentation liquid reached a peak at 18 h of fermentation, after centrifugal spray drying with the tower, the live bacterial biomass of the dry clay powder was lower than that of the powder obtained from the 24 h fermentation broth. Tian et al. (2005) examined the effect of temperature on the survival of six types of *Bacillus*. sp. and reported that temperatures above 60°C affect the survival of *Bacillus*. sp. The possible reason for the decrease in bacterial biomass after spray drying is that the formation rate and maturity of spores at 18 h were too low. Live bacteria that were not converted to spores were significantly lost during high-temperature spray drying at an inlet temperature of 175°C and an outlet temperature of 78°C–80°C. However, if the temperature is too low, the microbial modified clay powder will be too wet after spraying, which will affect the characteristics of the clay powder product (Qian et al., 2013). Therefore, 175°C and 80°C, which do not have a significant effect on spores, were chosen as the inlet and outlet temperatures, respectively, for our spray drying process. In contrast, although the biomass of live bacteria in the 24 h fermentation broth was less than that at 18 h, it remained high because of the relatively high spore formation rate and maturity rate. Therefore, after high-temperature powder spraying, the yield of live bacteria (including spores) from the liquid fermentation broth to the solid dry powder is higher.

The removal efficiency of the low-temperature freeze-dried material for red tide algae was not as good as that of the clay powder obtained from high-temperature centrifugal spray drying. Liquid nitrogen is often used in the food industry for quick freezing and preservation at low temperatures and is a common method of preserving pathogenic microorganisms (Cai et al., 2015). In the low-temperature environment, the metabolism or growth and reproduction of pathogenic microorganisms are inhibited, enabling long-term preservation of food or bacteria. Low-temperature freeze-drying had a certain killing effect on *Bacillus*. sp. in the modified clay fermentation broth, thereby reducing its removal efficiency against red tide organisms.

### 4.3 Analysis of the algae removal effect and mechanism of the composite microbial modified clay

In this study, the bacterial biomass of dry clay powder obtained at different fermentation times (6, 10, 18, and 24 h) was also positively correlated with the removal rate of *Heterosigma akashiwo*., similar to our previous lab study (Zhong et al., 2022). In the experiment with the addition of different clay concentrations (dose-effect experiment), the removal rate 3 h after the addition of microbial modified clay verified that the more microbial modified clay was used, the higher the removal rate of HAB organisms. Although a higher content or biomass of live bacteria is associated with a higher concentration of active algicidal substances produced and contained by the bacteria, the results confirm that the live bacterial biomass in the microbial modified clay is positively correlated with the effectiveness of red tide organism removal. Once the dosage reached the concentration threshold for eliminating *Heterosigma akashiwo*, a high removal effect was achieved. In the concentration gradient experiment, for 5 × 10^7^ cells/L *Heterosigma akashiwo*, the addition of 0.1 g/L microbial modified clay achieved a removal effect of more than 90%, corresponding to a threshold concentration of approximately 4.1 × 10^5^ cells/mL bacteria. This threshold is consistent with the threshold concentrations reported for previous algicidal experiments of 3.7 × 10^3^ to 1.3 × 10^6^ cells mL^–1^ (Seong and Jeong, 2013).

**FIGURE 6:**
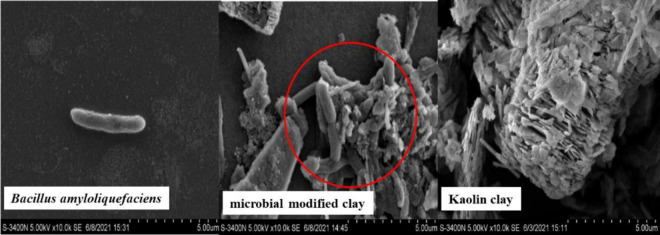
Electron microscopy images of clay and microorganisms in the composite fermentation process.

The surface of clay provides a necessary microenvironment for many microorganisms to conduct biogeochemical processes, and adsorption or adhesion between microorganisms and clay minerals is the basis of their interaction (Rong et al., 2008) (as shown in Figure 6). The presence of clay particles in the fermentation broth in this study may provide a suitable carrier and microenvironment for physiological and biochemical activities such as microbial proliferation, attachment or adhesion, and the secretion of active algicidal compounds. Therefore, the synergistic effects of both bacteria and algicidal substances in the microbial modified clay are important factors responsible for the superior removal effect of microbial modified clay compared with that of commercial bacteria or clays.

As mentioned above, microbial modified clay has multiple action mechanisms in the removal of red tide algae. That is, the removal effect of microbial modified clay on HAB organisms is a comprehensive result of the combined action of clay particles, microorganism cells, and algicidal substances. For inorganic modified clays (such as MCI and MCII used in this study), fast flocculation is the main mechanism (Yu et al., 1999), and their maximum effect can be reached in a short period of time, with no significant increase in removal rate in the later 3–24 h stage (Figure 2 right). For the microbial modified clay, although the removal rate at 3 h indicated that the removal effect was directly proportional to the concentration of microbial modified clay added, the removal rate obtained at almost all the dosages exceeded 90% after 24 h; moreover, there was no longer an obvious relationship with the added concentration after 24 h, as long as the threshold concentration was reached (Figure 4 left). The possible reason is that from 3 to 24 h, algicidal bacteria may proliferate in the algal solution, and algicidal active substances may be secreted and increase in content, resulting in a value that is no longer limited by the initial added dosage or concentration of microbial modified clay. Algicidal substances secreted by bacteria are currently considered the main mode of action by which most algicidal bacteria eliminate HABs (Huang et al., 2019). Even if the bacterial biomass of microbial modified clay obtained cannot exceed that of commercial bacteria (with a content of 100 billion per gram), the production of algicidal substances or algicides may be significantly higher in the presence of clay particles in the fermentation system than that in commercial products. A previous study showed that the main mode of action by which *Bacillus amyloliquefaciens* acts on HAB organisms is through the indirect killing effect of algicidal substances in laboratory conditions. Our experimental results also support this viewpoint (as shown in Figure 3). The algal removal effect of the bacterial and clay stock solution comes primarily from the filtrate, which is mainly composed of bacterial secretions obtained from the clay–bacterial co-fermentation broth. In this experiment, the removal effect of the bacteria and clay resuspension was not significant, especially at low dosages. Therefore, it can be preliminarily inferred that bacterial secretions (algicidal substances) of *Bacillus amyloliquefaciens* are the main component of the microbial modified clay stock solution responsible for removing red tide organisms. One concern that should be pointed out is that stability of algicidal compounds in natural environments may be poor. And the clays might act as a protector for these compounds to keep their activity longer in the field. So, it should be further explored the role of the clay particles in enhancing algicidal activity. A few specific experiments should be designed to explore: the adsorption of clay particles to both the bacterial cells and algicidal compounds to further investigate the interactions between these compounds, particles and cells in the future work.

Microbial modified clay powder obtained by fermentation for different times may contain different concentrations of algicidal substances that have indirect effects. Therefore, during composite fermentation with a mixture of both clay and microorganisms, an increase in fermentation time may enhance the intensity of interactions between microorganisms and clay particles. The addition of clay particles thereby results in a 1 + 1 > 2 removal effect by simultaneously increasing the number of microorganisms and the concentration of algicidal substances over time, whereas fermentation systems containing only the bacteria *Bacillus amyloliquefaciens* may have a lower removal effect, as proven by both our fermentation experiment and previous studies (Zhong et al., 2022). The detailed mechanism by which clay particles may enhance both the number of microorganisms and algicide excretion should be further studied in the future.

## 5 Conclusion and outlook

*Bacillus amyloliquefaciens*, a widely used microecological regulatory probiotic in eutrophic aquaculture water bodies, was fermented with green and natural clay materials to obtain microbial modified clay. During fermentation, clay particles may adsorb *Bacillus amyloliquefaciens* to serve as carriers and environments for their division, proliferation, and secretion of active algicidal substances. As the maturation time increases, the biomass of microorganisms may also increase, as well as the amount of algicidal substances; the associated mechanism strongly merits further study. The microbial modified clay powder has an appropriate water content, is easy to store for a long time, and efficiently removes algae. It exhibited a high elimination effect on typical HAB organisms compared with the effects of bacteria or clay alone. By combing the two aspects, both the function of emergency flocculation disposal (when HAB outbreaks) and long-term ecological regulation (to maintain a healthier phytoplankton community structure through specific algicidal effect) were achieved, resulting in a 1 + 1 > 2 effect when applied in the eutrophic aquaculture environment. These findings and advantages provide an important basis for further promoting the large-scale application of microbial modified clay in HAB control, toward a healthy aquaculture ecosystem.

From the perspective of applications, our present study is only a preliminary exploration of the translation of laboratory experiments to a pilot manufacturing process. Future work needs to address several other scientific issues, such as optimization of the fermentation formula, the quantitative contribution rate of active substances in the biological modified clay during the HAB removal process. Also, it is necessary to further study the adsorption thermodynamics between clay particles and microorganisms in the fermentation broth and the adsorption kinetics during fermentation to better understand the process and design the fermentation formula and operating parameters. Most importantly, extensive research on the ecological effects and subsequent scaling up the technology or further field trials, as well as the performance of microbial modified clay in eliminating different types of HABs should be conducted to further promote the translation of this technology from the laboratory to the field in the future. Now more and more aquaculture ponds industries in coastal areas have realized the importance of the water quality and the health of the aquaculture pond ecosystem. Faced with the eutrophic problems and disastrous HAB outbreaks, usage of microbial-modified clay in aquaculture would be a feasible way in both improving aquaculture ecosystem health and product of aquaculture organisms, exhibiting a bright economic and environmental benefits in the future.

## Data Availability

The datasets presented in this article are not readily available because some of the dataset could be provided not for commercial purpose. Requests to access the datasets should be directed to ZW, wuzx@qdio.ac.cn.

## References

[B1] Anderson D. M. Fachon E. Hubbard K. Lefebvre K. A. Lin P. Pickart R. (2022). Harmful algal blooms in the alaskan arctic an emerging threat as the ocean warms. *Oceanography* 35 130–139.

[B2] Cai Y. Yu Z. Zhao C. Yu D. Lu J. Qiu J. (2015). Preservation method and rejuvenation method of Paenibacillus polymyxa. *Acta Agric. Zhejiangensis* 27 75–79.

[B3] Cao X.-H. Song X. Yu M. Wang K. (2006). Mechanisms of removing red tide organisms by organo-clays. *Environ. Sci.* 27 1522–1530. 17111605

[B4] Chen H. (2018). Emergency treatment and reflection of red tide event of Gymnodinium catenatum in Fujian sea area in 2017. *J. Fish. Res.* 40 308–314.

[B5] Coyne K. J. Wang Y. Johnson G. (2022). Algicidal bacteria: A review of current knowledge and applications to control harmful algal blooms. *Front. Microbiol.* 13:871177. 10.3389/fmicb.2022.871177 35464927 PMC9022068

[B6] Demuez M. Gonzalez-Fernandez C. Ballesteros M. (2015). Algicidal microorganisms and secreted algicides: New tools to induce microalgal cell disruption. *Biotechnol. Adv.* 33 1615–1625. 10.1016/j.biotechadv.2015.08.003 26303095

[B7] Huang H. Han B. Zhang S. Wu F. (2019). Advance in marine algicidal bacteria research. *South China Fish. Sci.* 15 126–132.

[B8] Jeong S.-Y. Son H.-J. (2021). Effects of mycosubtilin homolog algicides from a marine bacterium, Bacillus sp. SY-1, against the harmful algal bloom species Cochlodinium polykrikoides. *Microbiol. J.* 59 389–400. 10.1007/s12275-021-1086-8 33779952

[B9] Jesús M. Liliana S. Łucja B. Sława G. Joanna M. (2024). Algicidal bacteria against cyanobacteria: Practical knowledge from laboratory to application. *Crit. Rev. Env. Sci. Tec.* 5 239–266.

[B10] Jung S. W. Kang H. Katano T. Kim H. Cho Y. Lee J. H. (2010). Testing addition of *Pseudomonas* fluorescens HYK0210-SK09 to mitigate blooms of the diatom Stephanodiscus hantzschii in small- and large-scale mesocosms. *J. Appl. Phycol.* 22 409–419.

[B11] Kang Y.-H. Jung S. W. Jo S.-H. Han M.-S. (2011). Field assessment of the potential of algicidal bacteria against diatom blooms. *Biocontrol Sci. Technol.* 21 969–984.

[B12] Li G. Xie G. Wang H. Wan X. Li X. Shi C. (2021). Characterization of a novel shrimp pathogen, Vibrio brasiliensis, isolated from Pacific white shrimp, Penaeus vannamei. *Fish. J. Dis.* 44 1543–1552. 10.1111/jfd.13475 34152602

[B13] Li Y. Lei X. Zhu H. Zhang H. Guan C. Chen Z. (2016). Chitinase producing bacteria with direct algicidal activity on marine diatoms. *Sci. Rep.* 6:21984. 10.1038/srep21984 26902175 PMC4763246

[B14] Liang Z. W. Shuo Z. Dawei Z. (2018). Research on physiological and biochemical index, growth and enzyme production characteristics of several *Bacillus strains*. *China Feed.* 11 85–88.

[B15] Liu C. Zhang L. Li Y. Hua G. Tian Y. Lin M. (2016). Optimization of sporulation medium of Bacillus subtilis B201. *Chin. J. Biol. Control* 32 650–656.

[B16] Liu S. Song X. Cao X. Yuan Y. (2021). Research on mitigation of prorocentrum donghaiense by micro-modified clay. *Oceanol. Limnol. Sin.* 52 1170–1179.

[B17] Liu Y. Cao X. Yu Z. Song X. Qiu L. (2016). Controlling harmful algae blooms using aluminum-modified clay. *Mar. Pollut. Bull.* 103 211–219. 10.1016/j.marpolbul.2015.12.017 26763322

[B18] Mayali X. Azam F. (2004). Algicidal bacteria in the sea and their impact on algal blooms. *J. Eukaryotic Microbiol.* 51 139–144. 10.1111/j.1550-7408.2004.tb00538.x 15134248

[B19] Meyer N. Bigalke A. Kaulfuss A. Pohnert G. (2017). Strategies and ecological roles of algicidal bacteria. *Fems Microbiol. Rev.* 41 880–899. 28961821 10.1093/femsre/fux029

[B20] Neves R. A. F. Nascimento S. M. Santos L. N. (2021). Harmful algal blooms and shellfish in the marine environment: An overview of the main molluscan responses, toxin dynamics, and risks for human health. *Environ. Sci. Pollut. Res.* 28 55846–55868. 10.1007/s11356-021-16256-5 34480308

[B21] Noh S. Y. Jung S. W. Kim B. H. Katano T. Han M.-S. (2017). Algicidal activity of the bacterium, *Pseudomonas fluorescens* SK09, to mitigate Stephanodiscus hantzschii (Bacillariophyceae) blooms using field mesocosms. *J. Freshw. Ecol.* 32 477–488.

[B22] Ouyang L. Liu Y. Chen H. Zaynab M. Yang X. Wang S. (2021). Encapsulation and algicidal properties of fermentation products from vibrio brasiliensis H115. *Front. Mar. Sci.* 8:676913. 10.3389/fmars.2021.676913

[B23] Pal M. Yesankar P. J. Dwivedi A. Qureshi A. (2020). Biotic control of harmful algal blooms (HABs): A brief review. *J. Environ. Manag.* 268:110687. 10.1016/j.jenvman.2020.110687 32383649

[B24] Qian J. Li S. Liu B. (2013). Research on mitigation of prorocentrum donghaiense by micro-modified clay. *Oceanol. Limnol. Sin.* 52 1170–1179.

[B25] Rediat A. Oon Y. L. Oon Y. S. (2024). Diverse interactions between bacteria and microalgae: A review for enhancing harmful algal bloom mitigation and biomass processing efficiency. *Heliyon* 10:e36503. 10.1016/j.heliyon.2024.e36503 39286093 PMC11402748

[B26] Rong X. Huang Q. Chen W. Wei L. (2008). Interaction mechanisms of soil minerals with microorganisms and their environmental significance. *Acta Ecol. Sin.* 28 376–387.

[B27] Seong K. A. Jeong H. J. (2013). Interactions between marine bacteria and redtide organisms in Korean waters. *Algae* 28 297–305. 10.4490/algae.2013.28.4.297

[B28] Tian Y. Yi H. Zhao Z. Wang A. (2005). Endurance of temperature ammonium hydroxide and ethanol by several strains of Bacillus. *Chem. Bioeng.* 7 38–39.

[B29] Wei Y. (2019). *Characterization of Salt Acclimated Strain of Bacillus Amyloliquefaciens PEBA20.* A Thesis for a Master’s degree. Shandong: Shandong Agricultural University.

[B30] Xie Z. F. Zhou K. Zhao Y. (2013). Optimization of fermentation parameters for Bacillus amyloliquefaciens HN with water purification. *Acta Agric. Boreali Sin.* 28 225–230.

[B31] Yu Z. Chen N. (2019). Emerging trends in red tide and major research progresses. *Oceanol. Limnol. Sin.* 50 474–486. 10.1016/S0140-6736(24)01822-1 39488222 PMC7616816

[B32] Yu Z. Xiaoxia S. Xiuxian S. Bo Z. (1999). Clay surfaces modification and its coagulation of red tide organisms. *Chin. Sci. Bull.* 44 617–620.

[B33] Yu Z. M. Song X. X. Cao X. H. Liu Y. (2017). Mitigation of harmful algal blooms using modified clays: theory, mechanisms, and applications. *Harmful Algae* 69 48–64. 10.1016/j.hal.2017.09.004 29122242

[B34] Zhang Y. Yu Z. Song X. Cao X. Liu Y. (2013). Study on removal of brown tideAureococcus anophagefferens by modified clay. *Acta Oceanol. Sin.* 35 197–203.

[B35] Zheng N. Ding N. Gao P. Han M. Liu X. Wang J. (2018). Diverse algicidal bacteria associated with harmful bloom-forming Karenia mikimotoi in estuarine soil and seawater. *Sci. Total Environ.* 632 1415–1420. 10.1016/j.scitotenv.2018.03.035 29727965

[B36] Zheng T. Lv J. Zhou Y. Su J. Yang X. Zhang J. (2011). Advance in study on microbial control of harmful algae blooms—-Exploitation and research on marine Algicidal bacteria. *J. Xiamen Univer. Nat. Sci.* 50 445–454.

[B37] Zhong Y. Yu Z. Liu S. Cao X. Song X. (2022). Removal effect of typical red tide organisms with bacillus and microbial modified clay. *Oceanol. Limnol. Sin.* 53 1089–1097.

[B38] Zhou J. Chen G. Zhu X. Chen L. Cai Z. (2014). A review of the relationship between algae and bacteria in harmful algal blooms. *Acta Ecol. Sin.* 34 269–281.

[B39] Zhou S. Yin H. Tang S. Peng H. Yin D. Yang Y. (2016). Physiological responses of *Microcystis aeruginosa* against the algicidal bacterium *Pseudomonas aeruginosa*. *Ecotoxicol. Environ. Saf.* 127 214–221. 10.1016/j.ecoenv.2016.02.001 26866757

